# Tidal volume significantly affects oxygenation in healthy pigs during high-frequency oscillatory ventilation compared to conventional ventilation

**DOI:** 10.1186/s12938-022-00984-x

**Published:** 2022-02-13

**Authors:** Karel Roubík, Jakub Ráfl, Martin Rožánek, Petr Kudrna, Mikuláš Mlček

**Affiliations:** 1 Department of Biomedical Technology, Faculty of Biomedical Engineering, Czech Technical University in Prague, nam. Sitna 3105, 272 01 Kladno, Czech Republic; 2 Institute of Physiology, First Faculty of Medicine, Charles University, Albertov 5, 128 00 Prague, Czech Republic

**Keywords:** Mechanical ventilation, High-frequency oscillatory ventilation, Oxygenation, Tidal volume

## Abstract

**Background:**

The role of high-frequency oscillatory ventilation (HFOV) has long been debated. Numerous studies documented its benefits, whereas several more recent studies did not prove superiority of HFOV over protective conventional mechanical ventilation (CV). One of the accepted explanations is that CV and HFOV act differently, including gas exchange.

**Methods:**

To investigate a different level of coupling or decoupling between oxygenation and carbon dioxide elimination during CV and HFOV, we conducted a prospective crossover animal study in 11 healthy pigs. In each animal, we found a normocapnic tidal volume (*V*_T_) after the lung recruitment maneuver. Then, *V*_T_ was repeatedly changed over a wide range while keeping constant the levels of PEEP during CV and mean airway pressure during HFOV. Arterial partial pressures of oxygen (P_a_O_2_) and carbon dioxide (P_a_CO_2_) were recorded. The same procedure was repeated for CV and HFOV in random order.

**Results:**

Changes in P_a_CO_2_ intentionally induced by adjustment of *V*_T_ affected oxygenation more significantly during HFOV than during CV. Increasing *V*_T_ above its normocapnic value during HFOV caused a significant improvement in oxygenation, whereas improvement in oxygenation during CV hyperventilation was limited. Any decrease in *V*_T_ during HFOV caused a rapid worsening of oxygenation compared to CV.

**Conclusion:**

A change in P_a_CO_2_ induced by the manipulation of tidal volume inevitably brings with it a change in oxygenation, while this effect on oxygenation is significantly greater in HFOV compared to CV.

## Background

In clinical practice, the elimination of carbon dioxide (CO_2_) during conventional mechanical ventilation (CV) depends on alveolar ventilation, defined as minute ventilation reduced by dead space ventilation. The effective removal of CO_2_ is therefore proportional to respiratory rate and tidal volume (*V*_T_). Oxygenation, on the other hand, is primarily controlled by the fraction of inspired oxygen (FiO_2_), mean airway pressure and positive end-expiratory pressure (PEEP) [1, 2]. Tidal volume, by opening lung alveolar units, also affects oxygenation [3], although *V*_T_ is not typically increased for this purpose because of concerns regarding ventilator-associated lung injury (e.g., volutrauma). The trial conducted by the ARDS Network [4] showed better oxygenation (but worse mortality) at *V*_T_ = 12 mL/kg versus *V*_T_ = 6 mL/kg.

One of the theoretical advantages of high-frequency oscillatory ventilation (HFOV) over other high-frequency modes is the relative decoupling of oxygenation and CO_2_ elimination [5, 6]. As with conventional mechanical ventilation, oxygenation during HFOV is primarily determined by mean airway pressure and by FiO_2_. Since tidal volume cannot be set directly on an adult HFOV ventilator, delivered tidal volume is set indirectly using pressure amplitude Δ*P* (controlled by the parameter called ‘power’) and the frequency of oscillations (Hz). *V*_T_ is increased by increasing Δ*P* (i.e., increasing ‘power’) or decreasing the frequency of oscillations [7]. At any given power and frequency combination, *V*_T_ delivery into the lungs is also influenced by relative inspiratory time (e.g., ‘% inspiratory time’, in fact inspiratory-to-expiratory ratio, I:E), and the diameter of the endotracheal tube (ETT). The portion of tidal volume delivered directly to the alveolar space may be further affected by the presence or absence of an ETT cuff leak [8–10]. In addition, the combination of a high Δ*P* and a low mean airway pressure (e.g., during weaning) may result in the entrainment of CO_2_ in the inspiratory limb of the HFOV ventilator circuit [11, 12]. Ventilation and thus CO_2_ elimination during HFOV are complex and determined by changes in power, oscillatory frequency, and by washout of CO_2_ around the endotracheal tube cuff.

When the authors started to use HFOV in adult patients with acute respiratory distress syndrome (ARDS) more than 15 years ago, they used a custom-made respiratory monitor for the measurement of *V*_T_ delivered during HFOV [13, 14]. It was noticed that *V*_T_ during HFOV was not only important for the control of CO_2_ elimination but that *V*_T_ affected oxygenation as well, even if mean airway pressure was kept constant. The authors also noticed that in contrast to CV, any degree of hyperventilation led to an appreciable increase in oxygenation. On the other hand, a slight decrease in ventilation, e.g., during permissive hypercapnia that is also recommended during HFOV [15–17], was associated with deterioration in oxygenation compared to the previous normocapnic period.

The aim of the study is to evaluate the effects of changes in tidal volume upon oxygenation during high-frequency oscillatory ventilation compared to conventional mechanical ventilation in healthy pigs.

## Results

A total of 11 healthy animals underwent the whole protocol of the study and corresponding data were used for evaluation. One additional animal died due to a cardiac arrest during the experiment. The animals were ventilated in the CV phase and in the HFOV phase. In each phase, we varied *V*_T_ over a wide range and recorded arterial partial pressures of oxygen (P_a_O_2_) and carbon dioxide (P_a_CO_2_). All data were aggregated into two groups corresponding to CV and HFOV. In total, the aggregated data contained 177 individual data entries (93 CV and 84 HFOV) containing *V*_T_, P_a_CO_2_ and P_a_O_2_ values.

**Fig. 1:**
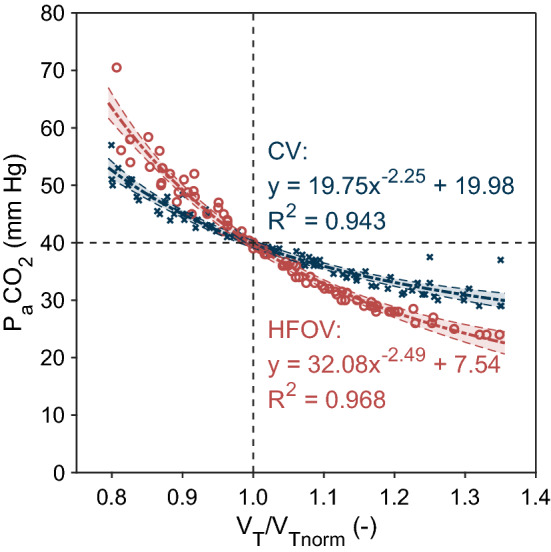
Stronger effect of tidal volume changes on CO_2_ elimination during high-frequency oscillatory ventilation (HFOV) compared to conventional mechanical ventilation (CV) close to normocapnia. Tidal volume is presented as relative to normocapnic tidal volume *V*_T norm_. Fitted curves are plotted with 95% functional prediction intervals

The effect of tidal volume changes on carbon dioxide elimination during both conventional and high-frequency ventilation is presented in Fig. 1. The power function $y=a{x}^{b}+c$ was applied to the HFOV data (*R*^2^ = 0.968) and to the CV data (*R*^2^ = 0.943). The results document a statistically significant (*P* < 0.001) stronger effect of tidal volume changes on CO_2_ elimination during HFOV compared to CV. The same relative change in tidal volume from the tidal volume that corresponds to normocapnia (P_a_CO_2_ = 40 mmHg) and is individual for each animal, affects the average level of P_a_CO_2_ more during HFOV than CV. For example, a decrease in tidal volume by 10% from normocapnia causes P_a_CO_2_ to increase by 5 mmHg (13%) on average during CV, but by 10 mmHg (24%) on average during HFOV. The increase in tidal volume by 10% from normocapnia reduces P_a_CO_2_ by 4 mmHg (10%) on average during CV, but by 7 mmHg (17%) on average during HFOV. As the ventilatory frequency was kept constant during the experiment, the minute ventilation was directly proportional to the delivered tidal volume; therefore, minute ventilation had a stronger effect on CO_2_ elimination in HFOV compared to CV.

**Fig. 2:**
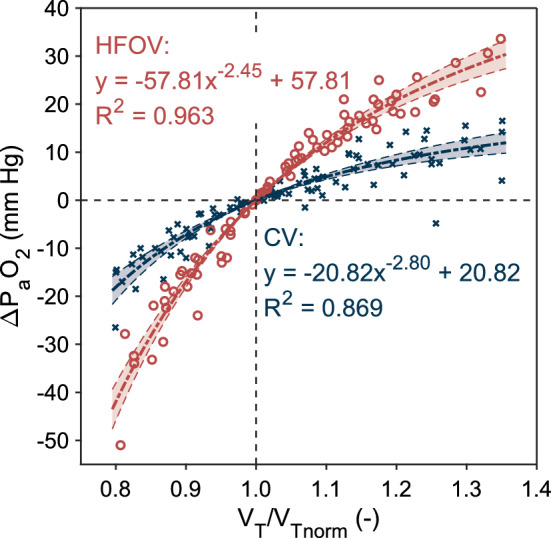
Stronger effect of tidal volume changes on oxygenation during high-frequency oscillatory ventilation (HFOV) compared to conventional mechanical ventilation (CV) close to the normocapnic P_a_O_2_ level. Tidal volume is presented as relative to normocapnic tidal volume *V*_T norm_. Fitted curves are plotted with 95% functional prediction intervals

The effect of changes in tidal volume on oxygenation is presented in Fig. 2. The same relative change in tidal volume, expressed as a ratio of the current and the normocapnic tidal volume, generates different changes in oxygenation during HFOV and CV. In this figure, the change in arterial partial pressure of oxygen (ΔP_a_O_2_) values represent differences in current P_a_O_2_ from the reference normocapnic P_a_O_2_ value determined for each animal separately. The power function $y=a{x}^{b}+c$ was applied to both the HFOV data (*R*^2^ = 0.963) and the CV data (*R*^2^ = 0.869). The function expresses the predicted average change in P_a_O_2_ in response to the change in tidal volume relative to the tidal volume that was titrated to achieve normocapnia. This effect of changes in tidal volume on the change of P_a_O_2_ is significantly stronger during HFOV (*P* < 0.001). Increasing tidal volume above its normocapnic value during HFOV represents a method to significantly improve oxygenation, whereas the improvement in oxygenation during CV hyperventilation is limited. Likewise, any decrease in tidal volume causes a rapid decrease in oxygenation during HFOV, whereas this effect is much smaller during CV. For example, the decrease in tidal volume by 10% from normocapnia causes an average reduction of P_a_O_2_ by 7 mmHg during CV, but by 17 mmHg during HFOV. The increase in tidal volume by 10% from normocapnia causes an average increase of P_a_O_2_ by 5 mmHg during CV, but 12 mmHg during HFOV. Additionally, as the ventilatory frequency was kept constant during the experiment, the same findings as those for tidal volume could be applied to minute ventilation.

**Fig. 3:**
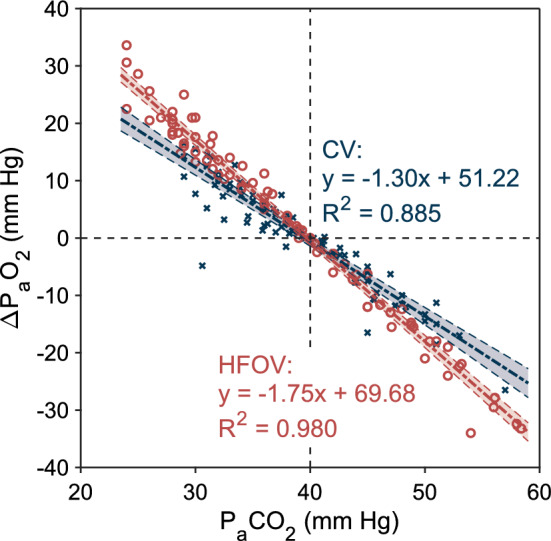
Comparison of changes in oxygenation relative to changes in P_a_CO_2_ during high-frequency oscillatory ventilation (HFOV) and conventional mechanical ventilation (CV). Fitted curves are plotted with 95% functional prediction intervals

Graph presented in Fig. 3 summarizes the previous two graphs (Figs. 1 and 2) and shows the different degree of interconnectedness of the changes in P_a_CO_2_ with the changes in P_a_O_2_ during CV and HFOV. The linear functions were fitted to the data (*R*^2^ = 0.980 for HFOV and *R*^2^ = 0.885 for CV). The graph documents that the change in P_a_CO_2_ induced by the manipulation of tidal volume inevitably brings with it a change in oxygenation (i.e., P_a_O_2_), while this effect on oxygenation is significantly greater in HFOV compared to CV (*P* < 0.001). As a result, for example, correction of P_a_CO_2_ by a change in *V*_T_ causes a significantly greater change in P_a_O_2_ in HFOV than in CV. The change in oxygenation in HFOV is more closely related to the change in P_a_CO_2_ than in CV.

## Discussion

The most important finding of this study is that changes in P_a_CO_2_ induced by adjustment of tidal volume during HFOV affect oxygenation significantly compared to the same P_a_CO_2_ changes during CV.

Furthermore, increasing tidal volume above its normocapnic value during HFOV represents a way of a significant improvement in oxygenation, whereas improvement in oxygenation during CV hyperventilation is limited.

The limitation of improving oxygenation while increasing tidal volumes in CV is well known [2, 18]. This means that increasing tidal volume is not considered as a tool to improve oxygenation during CV and the results of the current study are consistent with this principle. On the other hand, we did not find such a limitation in oxygenation improvement during HFOV. At the same time, any decrease in tidal volume causes a rapid worsening of oxygenation during HFOV, whereas this effect is much smaller during CV.

In order to decrease the tidal volume during CV, permissive hypercapnia may be introduced [15–17]. The reduction in oxygenation as an effect of the introduction of permissive hypercapnia during CV is unanticipated and has not been reported yet. The current study shows a stronger relationship between oxygenation and CO_2_ elimination caused by changes in tidal volume during HFOV. Thus, the use of permissive hypercapnia during HFOV may further deteriorate oxygenation. In practice, for example, there are cases when it is not possible to achieve normocapnia despite the use of the largest possible tidal volume, which is limited by setting the maximum possible value of the parameter ‘power’ determining the magnitude of pressure oscillations and thus the maximum achieved tidal volume. If hypercapnia is allowed in this case, worsened oxygenation can be expected as a consequence of the significant effect of the suboptimal tidal volume not only on the elimination of carbon dioxide but also on oxygenation. Permissive hypercapnia may also be instituted via intentional reduction of the pressure oscillations amplitude (by setting lower ‘power’) in order to strengthen the protectivity of HFOV to the lung tissue [19]. Intentional reduction of tidal volume in this scenario may negatively affect oxygenation as well. The authors speculate that in some cases the deterioration in oxygenation during HFOV with permissive hypercapnia may be falsely interpreted as HFOV inefficiency.

These results may help to explain the HFOV-related controversies after two large HFOV clinical studies OSCAR [20] and OSCILLATE [21] as well as the consequential analyses and review articles were published. Recent studies demonstrated that HFOV requires an individual approach when setting the ventilator [22, 23] and the lack of the individual approach to HFOV settings could have affected the outcome of the earlier large studies [24, 25]. Both in pediatric [26] and adult patients [27] HFOV needs to be applied on a case-by-case basis for which a better understanding of HFOV is required. The care of patients with ARDS is being further optimized, now also due to COVID-19 ARDS. Clinicians are studying new techniques and approaches. Effect of extracorporeal membrane oxygenation [28, 29], different levels of PEEP [30], prone positioning [31, 32], respiratory system impedance analysis [33], and different times of weaning [34] has been studied. For the use of HFOV in the treatment of ARDS, there is a need to understand the place of HFOV in the spectrum of diagnostic and therapeutic techniques for severe ARDS care and to define the optimal HFOV strategy [35]. For example, electrical impedance tomography seems to be a promising diagnostic tool for individualized settings of both HFOV [36] and CV [37].

During the study, we kept all the ventilatory parameters constant, except for the intentionally changed tidal volume (set indirectly using pressure amplitude Δ*P* during HFOV). If the intentional change in Δ*P* caused any change in other parameters, the setting of ventilator 3100B was immediately corrected. We especially observed mean airway pressure, as this parameter has a direct effect on oxygenation and therefore could affect the results of our study.

Several studies documented that mean alveolar pressure may differ from the mean airway pressure measured in the airway opening and measured by 3100B ventilator used. Pillow et al. [38] investigated, that I:E ratio is a crucial determinant of whether such a difference is present. Nevertheless, in their study, they found no evidence of significant air-trapping (i.e., the difference between mean alveolar pressure and mean airway pressure) at an I:E ratio of 1:1, which is the I:E ratio used in our study. Furthermore, they did not observe a significant development of air-trapping at I:E ratio of 1:1 across a wide range of Δ*P* from a minimum of 10 cmH_2_O to a maximum of 90 cmH_2_O. Several previous studies in dogs [39], rabbits [40], and adult human subjects [41, 42] have shown that air-trapping elevated alveolar pressure above mean airway pressure when HFOV was employed at an I:E ratio of 1:1. A number of mechanisms have been postulated to account for this effect, and each depends on the presence of asymmetry between inspiratory and expiratory resistance [38]. In our study, we did not have an opportunity to assess mean alveolar pressure and to confirm or disprove the presence of an air-trapping that could influence oxygenation. However, we do not consider this condition to be a disadvantage of our protocol as mean airway pressure measured at the airway opening is so far the only parameter used for setting mean airway pressure during HFOV in all the published studies.

Several reasons led us to choose a group of healthy animals for the study. As we planned to evaluate the effects of tidal volume changes during the course of each protocol phase, a model stable for several hours was required so that the recorded changes within a phase would be driven by *V*_T_ and would not be affected by changing lung condition and function. All ARDS models evolve in time and therefore the lung parameters change. The most common ARDS model induced by normal saline lung lavage does not represent a typical ARDS lung as ARDS in patients is rarely, if ever, solely a result of surfactant deficiency. Nevertheless, the effect of HFOV hyperventilation observed in this study in healthy animals corresponds to the results of an HFOV hyperventilation study conducted in a porcine ARDS model induced by saline lavage [43]. Hyperventilation with *V*_T_ increased to 135% of its normocapnic value increased P_a_O_2_ by 31 mmHg in the current study; whereas *V*_T_ increased to 146% caused an increase in P_a_O_2_ by 28 mmHg in the saline lavage ARDS model in pigs.

The intention of our study design was to approach, as much as possible, the way in which both the CV and HFOV are used in clinical practice. The ventilation modes differ in the mechanism of gas exchange, the way of control and the significance and typical values of their parameters. Thus, in attempting a fair comparison, we did not seek a formal agreement on some parameter of the ventilator setup such as the same initial tidal volume. Instead, we adopted the opposite approach, individualizing the ventilation settings to have the same resulting effect on the internal environment, i.e., normocapnia. The aim of the study was to show that the change of ventilation, that is used to adjust P_a_CO_2_, affects the P_a_CO_2_ and P_a_O_2_ differently during the CV and HFOV under the condition of unaltered PEEP and mean airway pressure, respectively. The role of the mean airway pressure in the ventilation control in HFOV can be viewed in a manner similar to the PEEP level in CV [44]. When a change in oxygenation is unnecessary, the parameters remain usually unchanged. Our study can be understood as a sort of sensitivity analysis, where we investigate how the relative change in tidal volume to an individually set normocapnic tidal volume affects the change in blood gases in HFOV compared to CV. Our results suggest that whereas during CV the changes in oxygenation due to changes in ventilation are more or less negligible, the relatively same percentual changes in ventilation during HFOV may affect oxygenation significantly and should be taken into account. During HFOV, ventilation and oxygenation are coupled more than it has been assumed so far. This difference in coupling of oxygenation and CO_2_ elimination is explicitly expressed by the different slopes of the CV and HFOV lines in Fig. 3, where P_a_O_2_ changes more rapidly for the same change in P_a_CO_2_ for HFOV than for CV.

## Conclusions

The relative change in tidal volume occurring during titration of a target P_a_CO_2_ during HFOV reasonably affects oxygenation during HFOV compared to CV. Increasing tidal volume improves oxygenation, whereas a decrease in tidal volume from its normocapnic value causes a rapid decrease in oxygenation during HFOV compared to CV. A change in P_a_CO_2_ induced by the manipulation of tidal volume inevitably brings with it a change in oxygenation, while this effect on oxygenation is significantly greater in HFOV compared to CV.

## Methods

The study protocol was approved by the Institutional Animal Care and Use Committee of the First Faculty of Medicine, Charles University. The study was performed in an accredited animal laboratory of the Institute of Physiology, First Faculty of Medicine, Charles University, in accordance with Act No. 246/1992 Coll., on the protection of animals against the cruelty that is harmonized with EU legislation.

Twelve crossbred (Landrace × Large White) female pigs (*Sus scrofa domestica*) 4–5 months old with an average body weight of 44 kg (42–54 kg range), were involved in the study.

### Anesthesia and preparation

Animals were premedicated by azaperone (2 mg/kg IM), followed by anesthesia with ketamine hydrochloride (20 mg/kg IM) and atropine sulphate (0.02 mg/kg IM). When placed on the operating table, initial boluses of propofol (2 mg/kg IV) and morphine (0.1 mg/kg IV) were administered. Animals were orotracheally intubated with a cuffed endotracheal tube (I.D. 7.5 mm) and connected to a conventional ventilator Hamilton G5 (Hamilton Medical AG, Bonaduz, Switzerland) in INTELLiVENT-ASV mode. An ear vein was cannulated and a continuous infusion of propofol (8 to 10 mg/kg/h IV) combined with morphine (0.1 to 0.2 mg/kg/h IV) and heparin (40 to 50 U/kg/h IV) was initiated to maintain anesthesia. The drugs doses were adjusted to keep the animals in the stage of surgical anesthesia throughout the procedure. The depth of anesthesia was checked according to reflex responses and brain monitoring of bispectral index (BIS). Initial rapid infusion of 1000 mL of normal saline was given intravenously, followed by a continuous IV infusion of 200 mL/h to 500 mL/h to reach and maintain central venous pressure of 5 to 7 mmHg. Femoral venous and arterial cannulation were performed to obtain central venous pressure (CVP, *v. femoralis*) and arterial blood pressure (ABP, *a. femoralis*). Cardiac output (CO), mixed venous blood oxygen saturation (SvO_2_) and pulmonary artery pressure (PAP) were measured continuously by a Swan–Ganz catheter inserted via the right femoral vein and connected to a hemodynamic monitor (Vigilance I, Edwards Lifesciences, Irvine, CA, USA). Arterial blood gases were monitored continuously using a continuous blood parameter monitoring system CDI 500 (Terumo, Tokyo, Japan), via a sensor placed in the extracorporeal arteriovenous shunt (between *a. femoralis* and *v. femoralis*). The muscle relaxant pipecuronium bromide (4 mg boluses every 45 min) was administered during the experimental phase to suppress spontaneous breathing.

### Study design

**Fig. 4:**
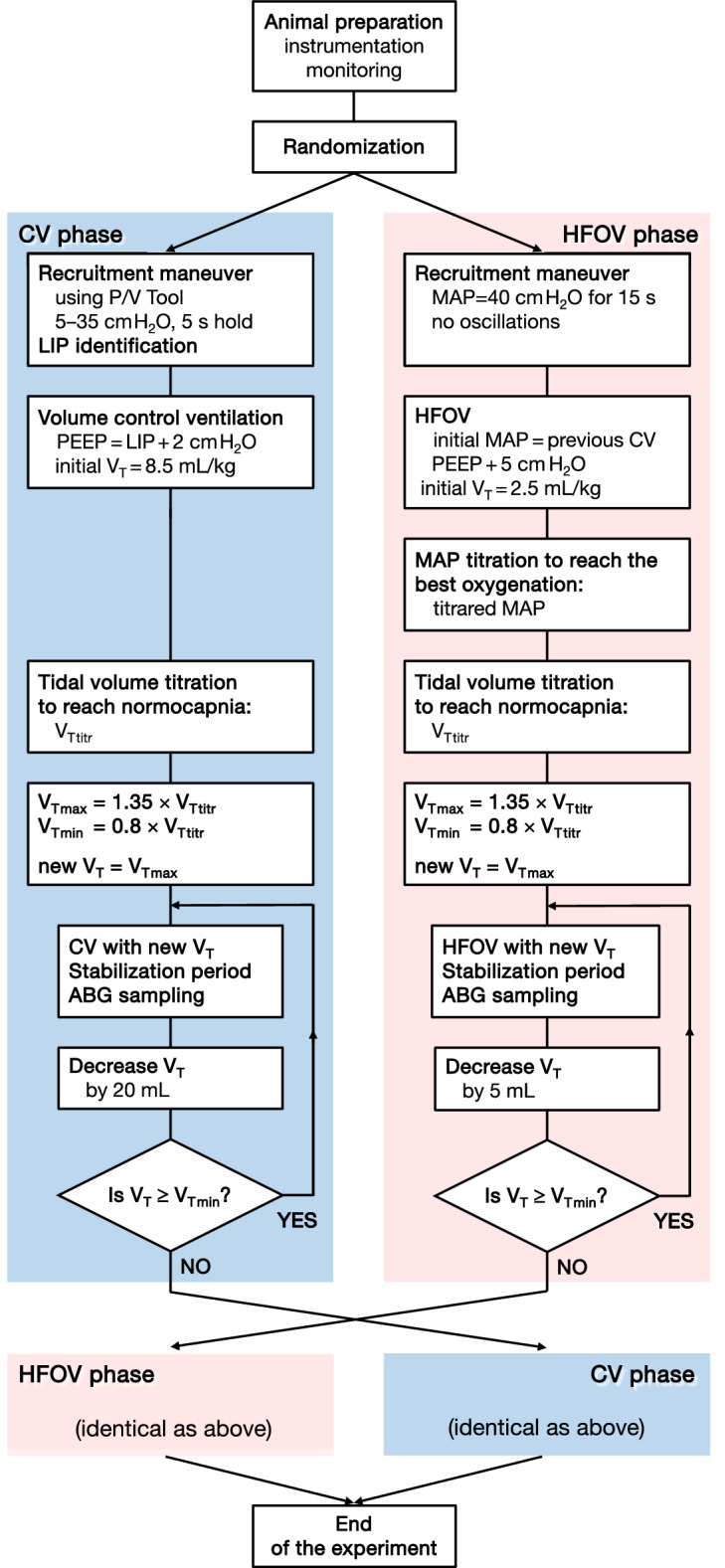
Summary of corresponding actions taken during the conventional mechanical ventilation (CV) phase and the high-frequency oscillatory ventilation (HFOV) phase of the crossover study. *MAP* mean airway pressure

The study was designed as a prospective interventional crossover study. The study protocol is summarized in Fig. 4. After the preparation, instrumentation, and muscle relaxation, the animals were randomized either to conventional ventilation (CV phase) or to high-frequency oscillatory ventilation (HFOV phase). The randomization was conducted using a table of random numbers. After the completion of the designated phase, the other phase was initiated immediately.

During the CV phase, pigs were ventilated using the conventional ventilator Hamilton G5 in the volume-controlled ventilation mode (synchronized controlled mandatory ventilation, (S)CMV). At the beginning of the phase, a recruitment maneuver was performed using the P/V Tool feature of the Hamilton G5 ventilator (increasing airway pressure from 5 to 35 cmH_2_O at the speed of 3 cmH_2_O/s and followed by an inspiratory hold for 5 s) in order to minimize effects of the lung volume history and conditions among the animals. The ventilator settings were: respiratory rate 18 breaths per minute, FiO_2_ 21%, I:E 1:1 and a decelerating flow pattern was selected. The positive end-expiratory pressure was set 2 cmH_2_O above the lower inflection point (LIP) of the static pressure–volume curve recorded using the P/V Tool provided by the ventilator [44, 45]. When PEEP predicted by the P/V Tool was lower than 5 cmH_2_O or the lower inflection point could not be identified, PEEP was set to 7 cmH_2_O. This ventilator setting, including FiO_2_, was kept constant during the whole CV phase. The initial tidal volume was set at 8.5 mL/kg of the actual body weight and was titrated to reach normocapnia (P_a_CO_2_ = 40 ± 1 mmHg) while maintaining a respiratory rate of 18 breaths per minute.

At the beginning of the HFOV phase, a recruitment maneuver was performed by inflating the lungs to a pressure of 40 cmH_2_O for 15 s [24] with no oscillations in order to minimize the effects of the lung volume history and conditions among the animals. A high-frequency oscillatory ventilator SensorMedics 3100B (SensorMedics, Yorba Linda, CA, USA) was used in the HFOV phase. The ventilator settings were: FiO_2_ 21%, bias flow 35 L/min, frequency of oscillations 5 Hz and I:E 1:1. The ventilator setting was kept constant during the whole HFOV phase. The HFOV mean airway pressure was initially set 5 cmH_2_O above the mean airway pressure during preceding conventional ventilation (CV phase or the preparatory phase when the animal was randomized to HFOV phase first) [5, 25]. Then, the mean airway pressure was titrated to reach the best oxygenation without compromising circulation. The increase of mean airway pressure was stopped when the heart rate reached 160 bpm, mean arterial blood pressure was equal to or less than 50 mmHg, or mean pulmonary artery pressure was equal to or greater than 35 mmHg. The initial tidal volume was set to 2.5 mL/kg of the actual body weight by adjusting Δ*P* on the ventilator and was titrated to reach normocapnia (P_a_CO_2_ = 40 ± 1 mmHg). There were no ETT cuff leaks used during HFOV.

The rest of both CV and HFOV phases were identical. The titrated normocapnic tidal volume *V*_T titr_ found at the beginning of each phase was used for determining the range of tidal volumes to be tested and was not used later for any adjustment or calculation. The maximum tidal volume *V*_T max_ was calculated as 135% of the normocapnic *V*_T titr_ whereas the minimum tidal volume *V*_T min_ was calculated as 80% of the normocapnic *V*_T titr_. First, *V*_T_ was set to the calculated *V*_T max_ and this setting was kept for at least 30 min. Arterial blood gases were recorded and their stability was confirmed after additional 5 min. Then, the tidal volume was reduced stepwise by 20 mL in CV phase or 5 mL in HFOV phase until *V*_T min_ was reached. After each change of *V*_T_, the ventilatory parameters were checked immediately. When the change of *V*_T_ induced a change in other parameters (e.g., mean airway pressure was slightly affected during HFOV), a correction was made to restore the original values recorded prior to the *V*_T_ change. Then, all the ventilatory parameters were kept constant for 15 min and arterial blood gases were recorded. After another 5-min period, the measurement was repeated. If the change in any of the arterial blood gases was not greater than 1 mmHg, the stepwise *V*_T_ reduction continued. Otherwise, another 5-min period for stabilization was introduced followed by another recording of arterial blood gases. The final values of blood gases at a given *V*_T_ were calculated as an average of the last two measurements. In some pigs it was not possible to achieve the calculated *V*_T max_ due to reaching maximum Δ*P* of the ventilator or to use the calculated *V*_T min_ due to severe hypoventilation of the animal. Blood oxygen saturation was continuously monitored, and the reduction of tidal volume was stopped if SpO_2_ fell below 85%. In such situations the range of set *V*_T_ was shortened accordingly.

Arterial blood gases (P_a_O_2_, P_a_CO_2_, and pH) were measured using the CDI 500 continuous blood parameter monitoring system. The accuracy of the system was verified prior to the experiment and then every 1 h by analysis of an arterial blood sample using a blood gas analyzer AVL Compact 3 (Radiometer, Copenhagen, Denmark). Heart rate, ABP, CVP, PAP, SpO_2_, ECG, body temperature, and BIS were monitored using the MU-631 RK (Nihon Kohden, Tokyo, Japan) patient monitor. CO and SvO_2_ were measured by the Vigilance I monitor.

As the SensorMedics 3100B HFOV ventilator does not measure tidal volume, the delivered tidal volume was measured using a Florian (Acutronic, Hirzel, Switzerland) respiratory function monitor. The same device was used to measure delivered tidal volume during CV in order to eliminate a systematic error. The Florian monitor was calibrated using a 1-L calibrating syringe (Hans Rudolph, Shawnee, KS, USA) and the accuracy of the monitor was also verified prior to the experiment using a VT Plus FH (Fluke Biomedical, Everett, WA, USA) gas flow analyzer during both CV and HFOV ventilatory modes.

### Data analysis and statistics

After the completion of each phase, characterized by the stepwise reduction of tidal volume from *V*_T max_ to *V*_T min_, a value of tidal volume *V*_T norm_ corresponding to normocapnia (P_a_CO_2 _= 40 mmHg) was determined and used for further calculations, regardless the titrated normocapnic tidal volume *V*_T titr_ found at the beginning of each phase. When the exact normocapnic P_a_CO_2_ level was not directly recorded in the measured data, *V*_T norm_ was estimated by linear interpolation between the two nearest tidal volumes. Then, relative tidal volumes, expressed as a percentage of *V*_T norm_, were calculated as *V*_T_/*V*_T norm_. The ventilation settings parameters (pressures and volumes) including the calculated *V*_T norm_ are summarized in Table 1.

**Table 1:** Ventilation settings and outcomes during the conventional ventilation (CV) phase and the high-frequency oscillatory ventilation (HFOV) phase of the experiment

Variable (unit)	CV	HFOV
PEEP (cmH_2_O)	9.3 ± 7.0	N/A
MAP (cmH_2_O)	13.1 ± 4.2	N/A
Initial MAP (cmH_2_O)	N/A	16.0 ± 3.8
Titrated MAP (cmH_2_O)	N/A	16.8 ± 4.0
*V*_T norm_ (mL)	371 ± 55	110 ± 12
*V*_T max_ (mL)	456 ± 78	129 ± 17
*V*_T min_ (mL)	317 ± 51	98 ± 14

Data are reported as mean ± standard deviation

*PEEP*, positive end-expiratory pressure; *MAP*, mean airway pressure; *V*_T norm_, tidal volume corresponding to normocapnia; *V*_T max_, maximum tidal volume; *V*_T min_, minimum tidal volume

The data obtained from all the animals were aggregated into two groups corresponding to CV and HFOV phases. Three relationships among the pooled data were examined: (1) The recorded P_a_CO_2_ levels related to the relative tidal volumes were analyzed. (2) The change in arterial partial pressure of oxygen (ΔP_a_O_2_) was calculated as the difference between P_a_O_2_ values and the normocapnic P_a_O_2_ (i.e., the level of P_a_O_2_ corresponding to *V*_T norm_). These ΔP_a_O_2_ values were analyzed with respect to relative tidal volumes. (3) Finally, the mutual relation between ΔP_a_O_2_ and P_a_CO_2_ as a result of changes in tidal volume was examined.

A power function in the form of $y=a{x}^{b}+c$ was used to fit each of the data groups (CV and HFOV) in the figures P_a_CO_2_ vs. *V*_T_/*V*_T norm_ and ΔP_a_O_2_ vs. *V*_T_/*V*_T norm_. The linear function was used to fit the data groups in the ΔP_a_O_2_ vs. P_a_CO_2_ figure. For the fitted curves, 95% functional prediction intervals were calculated. The curve fitting was performed in MATLAB Curve Fitting Toolbox (MathWorks Inc., Natick, MA, USA). The coefficient of determination (*R*^2^) was calculated for each of the fitted curves to evaluate the goodness of fit. For a pair of fitted curves in each of the figures, a version of the *F*-test applied to nonlinear models was used to determine whether the HFOV and CV trends differ significantly [46, 47]. *P* values less than 0.05 were considered as statistically significant.

## Data Availability

The datasets used and/or analyzed during the current study are available from the corresponding author on reasonable request.

## Abbreviations

ABP: Arterial blood pressure
ARDS: Acute respiratory distress syndrome
BIS: Bispectral index
CO: Cardiac output
CO_2_: Carbon dioxide
CV: Conventional mechanical ventilation
CVP: Central venous pressure
ECG: Electrocardiogram
ETT: Endotracheal tube
FiO_2_: Fraction of inspired oxygen
HFOV: High-frequency oscillatory ventilation
I:E: Inspiratory-to-expiratory ratio
IM: Intramuscular
IV: Intravenous
LIP: Lower inflection point
MAP: Mean airway pressure
P_a_CO_2_: Arterial partial pressures of carbon dioxide
P_a_O_2_: Arterial partial pressures of oxygen
PAP: Pulmonary artery pressure
PEEP: Positive end-expiratory pressure
*R*^2^: Coefficient of determination
(S)CMV: Synchronized controlled mandatory ventilation
SpO_2_: Peripheral oxygen saturation
SvO_2_: Mixed venous blood oxygen saturation
*V*_T max_: Maximum tidal volume
*V*_T min_: Minimum tidal volume
*V*_T norm_: Normocapnic tidal volume
*V*_T titr_: Titrated normocapnic tidal volume
*V*_T_: Tidal volume
Δ*P*: Pressure amplitude (‘power’)
ΔP_a_O_2_: Change in arterial partial pressure of oxygen
